# RIPK4 is associated with altered bioenergetics and invasive in melanoma three-dimensional models

**DOI:** 10.1038/s41598-026-59563-y

**Published:** 2026-06-23

**Authors:** Norbert Wronski, Jan Wolnik, Wacław Tworzydło, Aleksandra Bienia, Justyna Gogola-Mruk, Anna A. Brożyna, Anna Ptak, Agnieszka Wolnicka-Glubisz

**Affiliations:** 1 Department of Biophysics and Cancer Biology, Faculty of Biochemistry, Biophysics and Biotechnology, Jagiellonian University, Gronostajowa 7, Krakow, 30-387 Poland; 2 Doctoral School of Exact and Natural Sciences, Jagiellonian University, Kraków, Poland; 3 Department of Medical Biotechnology, Faculty of Biochemistry, Biophysics and Biotechnology, Jagiellonian University, Gronostajowa 7, Krakow, 30-387 Poland; 4 Department of Developmental Biology and Invertebrate Morphology, Institute of Zoology and Biomedical Research, Faculty of Biology, Jagiellonian University, Gronostajowa 9, Krakow, 30-387 Poland; 5 Laboratory of Physiology and Toxicology of Reproduction, Institute of Zoology and Biomedical Research, Faculty of Biology, Jagiellonian University, Gronostajowa 9, Krakow, 30-387 Poland; 6 Department of Human Biology, Institute of Biology, Faculty of Biological and Veterinary Sciences, Nicolaus Copernicus University, Lwowska 1, Toruń, 87-100 Poland; 7 Institute of Advanced Studies, Nicolaus Copernicus University in Toruń, Wileńska 4, Toruń, 87-100 Poland

**Keywords:** RIPK4, Melanoma, Metabolism, Bioenergetics, Invasion, Seahorse, Cancer, Cell biology, Molecular biology, Oncology

## Abstract

Melanoma is an aggressive cancer characterized by metabolic reprogramming that supports invasion and metastasis. Receptor-interacting protein kinase 4 (RIPK4) has been linked to tumor progression, but its role in melanoma metabolism remains unclear. This study examined whether RIPK4 is associated with melanoma aggressiveness through modulation of cellular bioenergetics. Previously generated RIPK4-knockout A375 and WM266.4 melanoma cell lines (CRISPR/Cas9) were used, and RIPK4 expression was restored by plasmid transfection. Cellular metabolism was assessed using the Seahorse XF Mito Stress Test in 2D monolayer cultures. In 3D spheroid models, ATP assays, qRT-PCR, and Western blotting were performed, together with functional analyses using 3D matrix invasion and CCID assays. In vivo relevance was evaluated by immunohistochemical analysis of RIPK4 and GLUT1 in lung metastases from NOD/SCID mouse xenografts. RIPK4 knockout induced a metabolically compromised state, characterized by reduced mitochondrial respiration and glycolysis, decreased HK2 and GLUT1 expression, and increased SDHB levels also impaired 3D invasive behavior, including reduced formation of invasive protrusions and decreased intravascular invasion. GLUT1 expression was detected in lung metastases but was reduced in RIPK4-deficient tumors. RIPK4 re-expression restored AKT phosphorylation and partially rescued HK2 and GLUT1 levels; however, metabolic flux (OCR/ECAR) was not recovered. These findings suggest that RIPK4 is associated with melanoma metabolic regulation and invasion, potentially involving AKT-linked signaling, but also indicate that additional mechanisms beyond AKT–GLUT1 contribute to the observed metabolic phenotype.

**Supplementary Information:**

The online version contains supplementary material available at https://doi.org/10.1038/s41598-026-59563-y.

## Introduction

Melanoma, a type of skin cancer originating from melanocytes, is known for its resistance to conventional therapies, highlighting the urgent need for novel treatment strategies. Despite significant advancements in targeted therapies and immunotherapy, the 5-year survival rate for patients diagnosed with melanoma exhibiting regional metastases remains below 75%, while it drops dramatically to approximately 28% in those with distant metastases^1^.

RIPK4 (Receptor-interacting serine/threonine-protein kinase 4) is known for its role in epidermal differentiation, but recent evidence has highlighted its emerging involvement in epithelial tumorigenesis^2^. Interestingly, its role in other cancers is complex and may depend on tissue type and biological context. While some studies have reported a decline in RIPK4 levels during tumor progression^3,4^, others have found its expression associated with more aggressive phenotypes and poor prognosis in several cancers, including ovarian and pancreatic cancer^5,6^.

Our previously published data, supported by in vivo experiments, demonstrated that RIPK4 knockout (RIPK4^KO^) cells formed significantly smaller tumors with delayed growth following subcutaneous implantation^7^. Moreover, tail vein injection of RIPK4^KO^ cells in mice resulted in fewer and smaller metastatic foci in the lungs^8^. These findings indicate that RIPK4 downregulation suppresses melanoma growth and metastasis in xenograft models. Our recent studies further support an oncogenic role for RIPK4 in melanoma, providing evidence that it contributes to disease progression through the activation of multiple signaling pathways, including NF-κB, Wnt/β-catenin, and FAK/AKT^7,9–11^.

On the other hand, it has been described that these pathways are crucial for various cellular processes, including metabolism^12^. Fundamental biological processes such as energy production, biosynthesis, and cell signaling, which are essential for cellular growth and adaptation to environmental stimuli, are tightly regulated by cellular metabolism. In melanoma - the most aggressive form of skin cancer - metabolic reprogramming plays a pivotal role by facilitating uncontrolled proliferation, enhanced survival, and resistance to therapeutic interventions. These changes involve a transformation in energy consumption patterns and nutrient acquisition strategies, conferring a survival advantage and promoting tumor progression, including invasion, immune evasion, and therapy resistance^13^. However, there is a lack of data on the direct involvement of RIPK4 in the regulation of melanoma cell metabolism. Moreover, RIPK4 knockdown was shown to inhibit epithelial-mesenchymal transition (EMT) by inactivating the Wnt/β-catenin pathway, suggesting a potential role in metabolic regulation through this pathway^12^. Given the centrality of altered metabolism in melanoma progression, investigating the molecular mechanisms that drive metabolic changes in metastatic melanoma is critical for the development of effective, targeted therapeutic strategies.

To address this, we used A375 and WM266.4 melanoma cell lines with stable RIPK4 knockdown (CRISPR/Cas9) and analyzed metabolic changes in a combination of 2D and 3D models mimicking in vivo conditions. Using 3D spheroid cultures, we assessed glucose uptake, the expression of metabolism-related genes and proteins, mitochondrial function (ATP assays), and mitochondrial morphology (TEM). Mitochondrial respiration was additionally analyzed using the Seahorse XF system in 2D monolayer cultures. Invasiveness was evaluated through formation of invasive protrusions and the CCID assay. These complementary approaches provided an integrated analysis of how RIPK4 influences energy metabolism and invasive potential in melanoma cells. Our results showed that RIPK4 deficiency leads to a metabolically compromised state including impaired mitochondrial function, reduced glucose uptake, and diminished invasive capacity. Taken together, these findings highlight a potential role for RIPK4 in the metabolic regulation of melanoma and suggest its relevance as a candidate target for further investigation.

## Materials and methods

### Chemicals

Methylcellulose and TIMP-1 ELISA were from Bio-Techne (McKinley Pl NE, MN, USA). FBS, CellTracker Green CMFDA, all TaqMan primers, TaqMan Array Human Extracellular Matrix & Adhesion Molecules Array, Geltrex™ LDEV-Free hESC-Qualified Reduced Growth Factor Basement Membrane Matrix and TaqMan™ Gene Expression Cells-to-CT™ Kit were from Thermo Fisher Scientific (Waltham, MA, USA). CellTiter-Glo^®^ 3D Cell Viability Assay, Glucose Uptake-Glo™ Assay were from Promega (Wisconsin, USA). Rabbit antibodies: anti-RIPK4, anti-GLUT1, anti-HK2, anti-IDH2, anti-SDHB, anti-CYCLIN D1, anti- CD44, anti-GAPDH and goat HRP-conjugated anti-rabbit IgG were from Cell Signalling Technology (Danvers, USA). RPMI-1640 culture medium with L-glutamine was from Sartorius (Kostrzyn, Poland). The 12% Bis-Tris gels - TGX™ FastCast™ Acrylamide Kit and Clarity Western ECL substrate were from Bio-Rad (Hercules, CA, USA). PVDF membranes were from Merck Millipore (Darmstadt, Germany). MV2 medium with the supplementation was from PromoCell GmbH (Heidelberg, Germany). Methanol was purchased from POCH (Poland). Seahorse Mito Stress, XF calibrant, pyruvate, glucose and glutamine were from Agilent (Santa Clara, CA, USA). Protease inhibitor cocktail, RIPA buffer, Bicinchoninic Acid Protein Assay Kit (BCA), penicillin 150 U/mL, and streptomycin 100 µg/ml, glutaraldehyde, formaldehyde were from Sigma-Aldrich (St. Louis, MO, USA). qPCR-HS Mix Probe was from A&A Biotechnology (Gdansk, Poland). Bovine serum (BSA) was from BioShop (Burlington, ON, Canada).

### Cell culture

Human melanoma cell lines A375 (ATCC, Manassas, VA, USA; #CRL-1619, purchased in 2021) and WM266.4 (Rockland, Limerick, PA, USA; #WM266-4-01-0001, purchased in 2025), along with phenotypically stable sub-lines of A375 and WM266.4 with varying degrees of RIPK4 downregulation (A375^RIPK4.KO^ and WM266.4^RIPK4.KO^) and their RIPK4 knockout-negative (neg) counterparts, as previously described^7^, were used in this study. A375 was authenticated using STR profiling in 2025 at the Genomics Core Facility, Jagiellonian University. Analysis included 14 standard loci including Amelogenin, and the resulting profiles were compared to reference profiles to confirm cell line identity. Melanoma cells were cultured in RPMI-1640 medium with L-glutamine (#01-100-1 A) supplemented with 10% FBS and 1% antibiotics (penicillin 150 U/ml, streptomycin 100 µg/ml).

Primary human aortic endothelial cells (HAEC, ATCC #PCS-100-011), donated by prof Łoboda (from Dept. Medical Biotechnology, UJ) were cultured in Endothelial Cell Medium MV2 supplemented with a specific cocktail of growth factors and other components. The medium included Epidermal Growth Factor (EGF) at 5 ng/ml, Basic Fibroblast Growth Factor (bFGF) at 10 ng/ml, Insulin-like Growth Factor (Long R3 IGF) at 20 ng/ml, and Vascular Endothelial Growth Factor 165 (VEGF 165) at 0.5 ng/ml. Additionally, the medium contained Ascorbic Acid at 1 µg/ml, Hydrocortisone at 0.2 µg/ml, and 5% Fetal Calf Serum (FCS). The cells were incubated at 37 °C in a 95% air/ 5% CO_2_ humidified incubator. All cell lines were routinely tested for mycoplasma contamination every six months.

### Spheroid formation

Spheroids were created using the hanging drop method as previously described^8,14^. Briefly, A375^RIPK4.KO^, WM266.4^RIPK4.KO^ and their negative counterparts (6 × 10^3^ cells per spheroid) were suspended in medium supplemented with 10% FBS and methylcellulose at a final concentration of 0.35% (#HSC011). After 4 days of incubation, spheroids were imaged using an OPTIKA IM-3 microscope (OLYMPUS, IX73, Japan) and their morphology was analyzed using a custom ImageJ macro designed for spheroid analysis.

### 3D matrix invasion assay

A 96-well plate was coated with 50 µl of Geltrex™ LDEV-Free, hESC-Qualified, Reduced Growth Factor Basement Membrane Matrix (#A1413302) per well and incubated at 37 °C for 1 h to allow gel polymerization. Spheroids were then carefully transferred to the wells, followed by the addition of 20 µl of Geltrex and 100 µl of complete medium. Images were captured at 0 and 24 h using the JuliStage imaging system (NanoEnTek Inc., Seoul, South Korea) at 10× magnification to monitor formation of invasive protrusions.

### CCID assay

HAEC monolayers were seeded into 96-well plates in MV2 complete medium. After 24 h, WM266.4 and A375 wild-type (WT) and RIPK4^KO^ spheroids were transferred onto CellTracker-stained HAEC cells (#C7025, 5 µM). After co-incubation for 0 and 3 h, cell-free areas formed in the Cytotracker (green)-stained HLEC monolayer directly beneath the melanoma spheroids were visualized using a bright-field/fluorescence microscope equipped with an Axiocam 503 camera (Zeiss, Oberkochen, Germany). CCID areas were quantified using ImageJ software (National Institutes of Health, USA).

### Glucose Uptake and ATP Quantification in 3D Spheroid Cultures

Glucose uptake in 3D spheroid cultures was assessed using the Glucose Uptake-Glo™ Assay Kit (#J1341) according to the manufacturer’s instructions. Briefly, spheroids together with the droplet-forming medium were transferred into a white 96-well plate and washed with phosphate-buffered saline (PBS). They were then incubated with 1 mM 2-deoxyglucose (2-DG) for 10 min at room temperature. Glucose uptake was terminated by the addition of acid detergent solution, followed by neutralization with Neutralization Buffer. Subsequently, 100 µl of 2DG-6-P Detection Reagent was added to each well, and luciferase activity was measured after 1 h using a CLARIOstar microplate reader (BMG Labtech, Germany).

ATP levels were determined using the CellTiter-Glo^®^ 3D Cell Viability Assay (#G9681), following the protocol described by Olajossy et al.^14^. Luminescence was measured using the same CLARIOstar reader (BMG Labtech, Germany).

### Transmission Electron Microscopy (TEM) Sample Preparation

The spheroids were fixed in a mixture of 2.5% glutaraldehyde and 1.5% formaldehyde in 0.1 M phosphate buffer for several weeks. The material was then postfixed in a solution of 1% osmium tetroxide and 0.8% potassium ferrocyanide for 60 min at 4 °C. After dehydration in the graded series of ethanol and acetone, the material was infiltrated in a mixture of acetone and epoxy resin, placed in a vacuum drier (Thermo Fisher Scientific, Waltham, Massachusetts, USA) for three hours and embedded in epoxy embedding resin kit (Merck Life Science). The resulting blocks were sectioned on an ultramicrotome into 70–80 nm ultrathin sections. The sections were contrasted according to standard procedures (with uranyl acetate and lead citrate) as described in Reynolds 1963^15^ and analyzed using a transmission electron microscope TEM (Jeol JEM 2100).

### mRNA Isolation and TaqMan Array Analysis

Total RNA was isolated from six spheroids per sample using the TaqMan™ Gene Expression Cells-to-CT™ Kit (#4399002), according to the manufacturer’s protocol. Reverse transcription was performed in a final volume of 50 µl using a ThermoMixer C thermoblock (Eppendorf, Germany). After completion of the reaction, samples were diluted with sterile water to a final volume of 150 µl.

For gene expression profiling, the TaqMan^®^ Array Human Extracellular Matrix & Adhesion Molecules (#4414133) was used. Each well received 10 µl of cDNA and 10 µl of qPCR-HS Mix Probe (#2008HS-1000P, A&A Biotechnology). The array plate, pre-loaded with TaqMan probes, was sealed with adhesive film, centrifuged, and subjected to real-time PCR using a qTOWER^3 thermal cycler (Analytik Jena, Germany). Transcript levels were calculated using the 2^−ΔΔCt^ method, with GAPDH used as the endogenous control.

Real-time PCR analysis of gene expression levels such as: N-cadherin (CDH2; Hs00983056_m1), GAPDH; Hs99999905_m1, MPC2; Hs00967250_m1, DLST; Hs04276516_g1, SDHA; Hs00417200_m1, PKM; Hs00761782_s1, PFKM; Hs01075411_m1, LDHA; Hs01378790_g1, FH; Hs00264683_m1, IDH1; Hs00271858_m1, IDH2; Hs00953879_m1, CS; Hs02574374_s1, ACO2; Hs00426616_g1, MPC1; Hs00211484_m1, HK1; Hs00175976_m1, GLUT1 (SLC2A1; Hs00892681_m1), GLUT4 (SLC2A4; Hs00168966_m1), HK2; Hs00606086_m1, were measured by real-time PCR as described in our previous study^9^. All expression levels were normalized to GAPDH and calculated using the 2^-ΔΔCt^ method^16^.

### Western blot

Ten spheroids per condition were lysed in RIPA lysis buffer (#R0278, Sigma-Aldrich). Protein concentrations were determined using a bicinchoninic acid (BCA) protein assay kit (#B9643 , Sigma-Aldrich), following the manufacturer’s instructions. Prior to hybridization, membranes were cut and incubated with the following human-specific primary antibodies: RIPK4 (#12636), CYCLIN D1 (#55506), GLUT1 (#12939), IDH2 (#56439), SDHB (#92649), HK2 (2867), CD44 (#3570), pAKT (#4060) and AKT (#4691). GAPDH (#5174) was used as a loading control. Protein expression in melanoma spheroids was analyzed by Western blotting as described in our previous study^17^. Immunopositive bands were visualized using the Clarity™ Western ECL substrate (#1705060) and detected with a ChemiDoc imaging system (Bio-Rad, Hercules, CA, USA). Band intensities were quantified using ImageLab software (version 5.2.1, Bio-Rad). Original, uncut, and unprocessed scans of all blots are provided in Supplementary Fig.S1.

### Seahorse XF Mito Stress Test

The day before the measurement, the Extracellular Flux Cartridge (Agilent) was rehydrated by adding 200 µl of XF Calibrant (#103059-000, Agilent Technologies) to each well and incubated overnight at 37 °C in a CO₂-free incubator. A total of 8 × 10^3^ cells per well were seeded onto a Seahorse XF Cell Culture Miniplate in standard growth medium, with the exception of background wells (A and H), which were left cell-free. The plate was incubated overnight at 37 °C with 5% CO_2_. The next day, the medium was removed and each well was washed with 200 µl of Seahorse XF DMEM assay medium, pH 7.4, supplemented with 1 mM pyruvate, 10 mM glucose, and 2 mM glutamine. Subsequently, 180 µl of the same assay medium was added to each well, and the plate was incubated for 1 h at 37 °C in a CO₂-free incubator to allow for temperature and pH equilibration.

Thirty minutes before the end of plate incubation previously rehydrated Extracellular Flux cartridge was prepared. Port A was filled with 20 µl, 10 µM oligomycin, port B with 22 µl 10 µM FCCP, and port H with 25 µl 10 µM rotenone and 10 µM antimycin. The XF Cell Mito Stress Test (#103015-100, Agilent Technologies) was performed using the Seahorse XFp Analyzer with default settings, following the manufacturer’s protocol. Data were normalized to total protein content and expressed as oxygen consumption rate (OCR) and extracellular acidification rate (ECAR) per 1 µg of protein. For clarity, temporal OCR/ECAR profiles were normalized to baseline for visualization, whereas all quantitative Seahorse parameters were calculated from absolute values and normalized to total protein content.

### Determination of TIMP-1 secretion by ELISA

The concentration of secreted TIMP-1 was measured in conditioned media using a human TIMP-1 Quantikine ELISA Kit ( #DTM100, R&D Systems, Bio-Techne), according to the manufacturer’s instructions. Conditioned media were collected and pooled from 15 spheroids per sample. After media collection, spheroids were lysed in RIPA buffer supplemented with protease inhibitors, and total protein concentration was determined using the BCA assay kit. TIMP-1 secretion levels were normalized to the total protein content of the corresponding spheroid lysates.

### Immunohistochemistry (IHC) for GLUT1 and RIPK4 expression

Formalin-fixed paraffin-embedded (FFPE) lung tissue sections used in this study originated from previously described melanoma metastasis experiments^8^. Briefly, mice were euthanized by an overdose of a ketamine/xylazine (150 mg /40 mg/kg). Following administration, animals were deeply anesthetized, as confirmed by loss of consciousness and absence of pain reflexes. Death was subsequently confirmed by exsanguination via cardiac puncture, followed by thoracotomy and collection of blood and tissue samples. Animal experiments were approved by the II Local Ethics Committee of the Institute of Pharmacology of the Polish Academy of Sciences (approval numbers: 82/2023, 135/2023, and 52/2024). All procedures were performed in accordance with relevant institutional and national guidelines and regulations and complied with the ARRIVE guidelines for reporting animal research.

GLUT1 and RIPK4 expression was assessed immunohistochemically in metastatic lesions in lung tissue samples obtained from NOD/SCID (non-obese diabetic severe combined immunodeficiency) mice. RIPK4 previously stained^8^ followed established protocols^9,10^. GLUT1 expression was assessed to evaluate glucose transporter levels in metastatic melanoma cells, according to the following protocol. After deparaffinization and rehydration, the sections were treated with a low-pH buffer (Antigen Unmasking Solution, cat. H-3300, Vector Laboratories) for antigen retrieval. Primary monoclonal antibody (GLUT1, E4S6I, Rabbit mAb, 73015 S, Cell Signaling Technology) was applied at a 1:250 dilution and incubated overnight at 4 °C. Visualization was performed using a secondary antibody conjugated to peroxidase (ImmPRESS^®^ Goat Anti-Rabbit IgG Polymer Kit, Peroxidase, MP-7451, Vector Laboratories) and a peroxidase substrate (ImmPACT^®^ NovaRED^®^ Substrate Kit, Peroxidase, cat. SK-4805, Vector Laboratories), according to the vendor’s protocol. After counterstaining of cell nuclei with hematoxylin (cat. 124687402, CHEMPUR), the sections were dehydrated, cleared in xylene, and mounted with VectaMount™ permanent mounting medium (cat. H-5000, Vector Laboratories).

Sections were coded and scored in a blinded manner. Staining intensity (SI) was assessed using a 4-point scale, from 0 to 3, with 0 as negative (0), weak (1), moderate (2), and strong (3) in relation to the positive control, respectively, pancreas for RIPK4 and placenta for GLUT1. In addition, the percentage of immunoreactive cells was also assessed (IR). Staining intensity and percentage of immunoreactive cells were evaluated manually in a blinded manner after assessment the data were paired with sample number. The whole section was evaluated. Sections were additionally counterstained with haematoxylin.

### RIPK4 Rescue in Knockout cells

RIPK4 expression was reintroduced in A375^RIPK4.KO^ cells by transfection with a RIPK4-expressing plasmid, pRP(Exp)_EGFP/Neo-CMV> hRIPK4/FLAG, or an empty vector control, pRP(Exp)-CMV> EGFP (VectorBuilder Inc., Chicago, IL, USA) using Lipofectamine™ 2000 (Thermo Fisher Scientific, Waltham, MA, USA), following Wroński et al., 2026^8^. Briefly, 2 × 10⁵ cells were seeded 24 h before transfection. Plasmid DNA and Lipofectamine 2000 were separately diluted in Opti-MEM, incubated, combined to form complexes, and added to cells. After 24 h, the medium was replaced with complete culture medium, and cells were cultured for another 24 h. Successful RIPK4 expression was confirmed by GFP fluorescence and Western blotting.

### Statistical analysis

Data are presented as mean ± SD, unless otherwise specified in the figure legends. Statistical tests used are indicated in figure legends. Analyses were performed using GraphPad Prism software (version 9.0; GraphPad Software, La Jolla, CA, USA). Statistical significance was determined using a two-tailed unpaired Student’s *t*-test. Statistical significance was defined as *p* < 0.05 (*), *p* < 0.01 (**), *p* < 0.001 (***), and *p* < 0.0001 (****), consistent with the notations used in the figures.

## Results

### The invasion and intravasation of two human melanoma cell lines with distinct metastatic potential is impaired by knocking out RIPK4

**Fig. 1:**
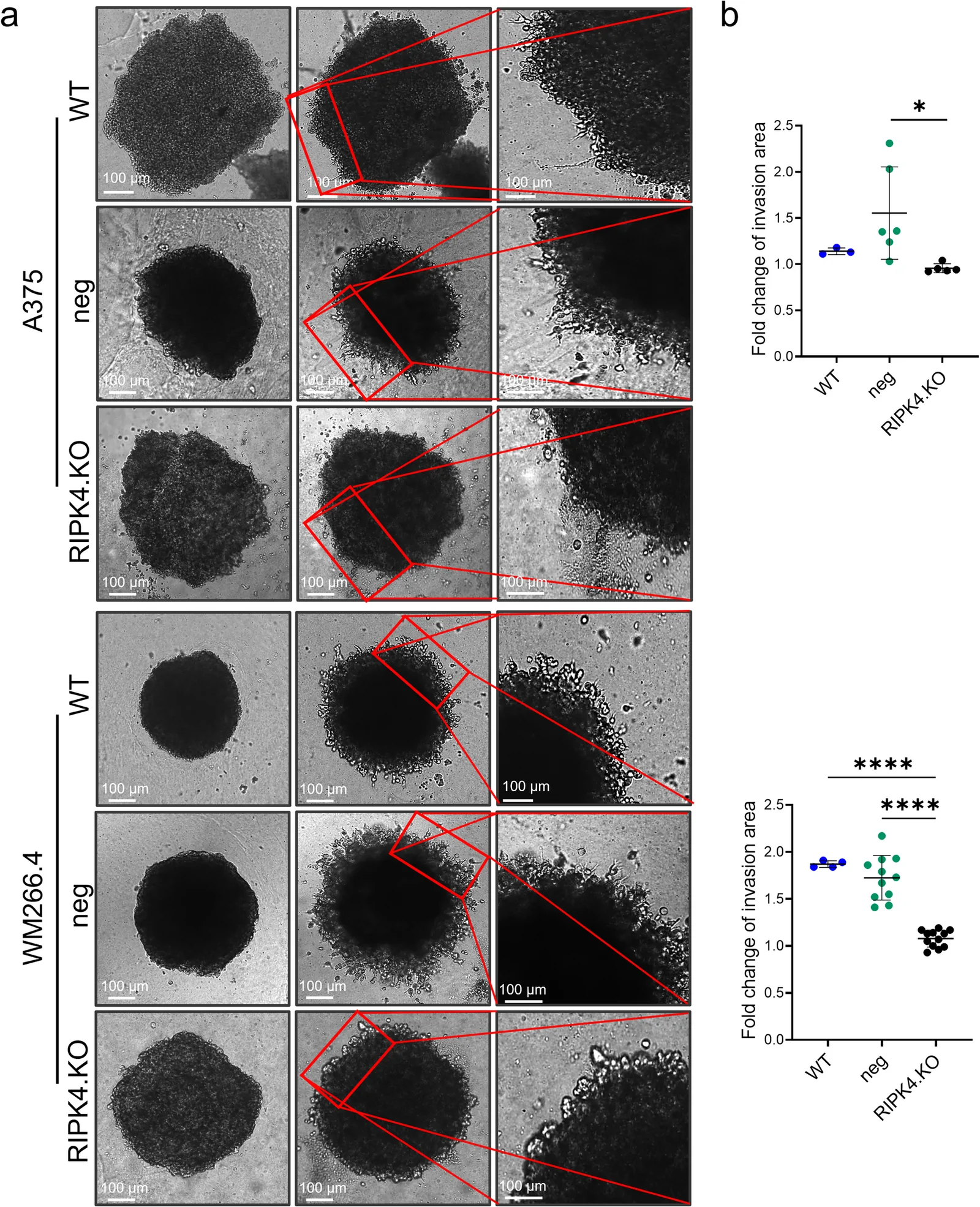
3D matrix invasion in A375 and WM266.4 melanoma spheroids following RIPK4 downregulation. (**a**) Representative images of spheroids A375^RIPK4.KO^, WM266.4^RIPK4.KO^ cells and their negative (neg) and wild-type (WT) controls at 0 and 24 h. (**b**) Quantification of the invasion area (A375, *n* = 3–6; WM266.4, *n* = 4–12). Data represent the mean ± SD from three independent experiments. Statistical analysis was performed using a two-tailed unpaired Student’s *t*-test. Statistically significant differences are denoted as * *p* < 0.05, **** *p* < 0.0001.

**Fig. 2:**
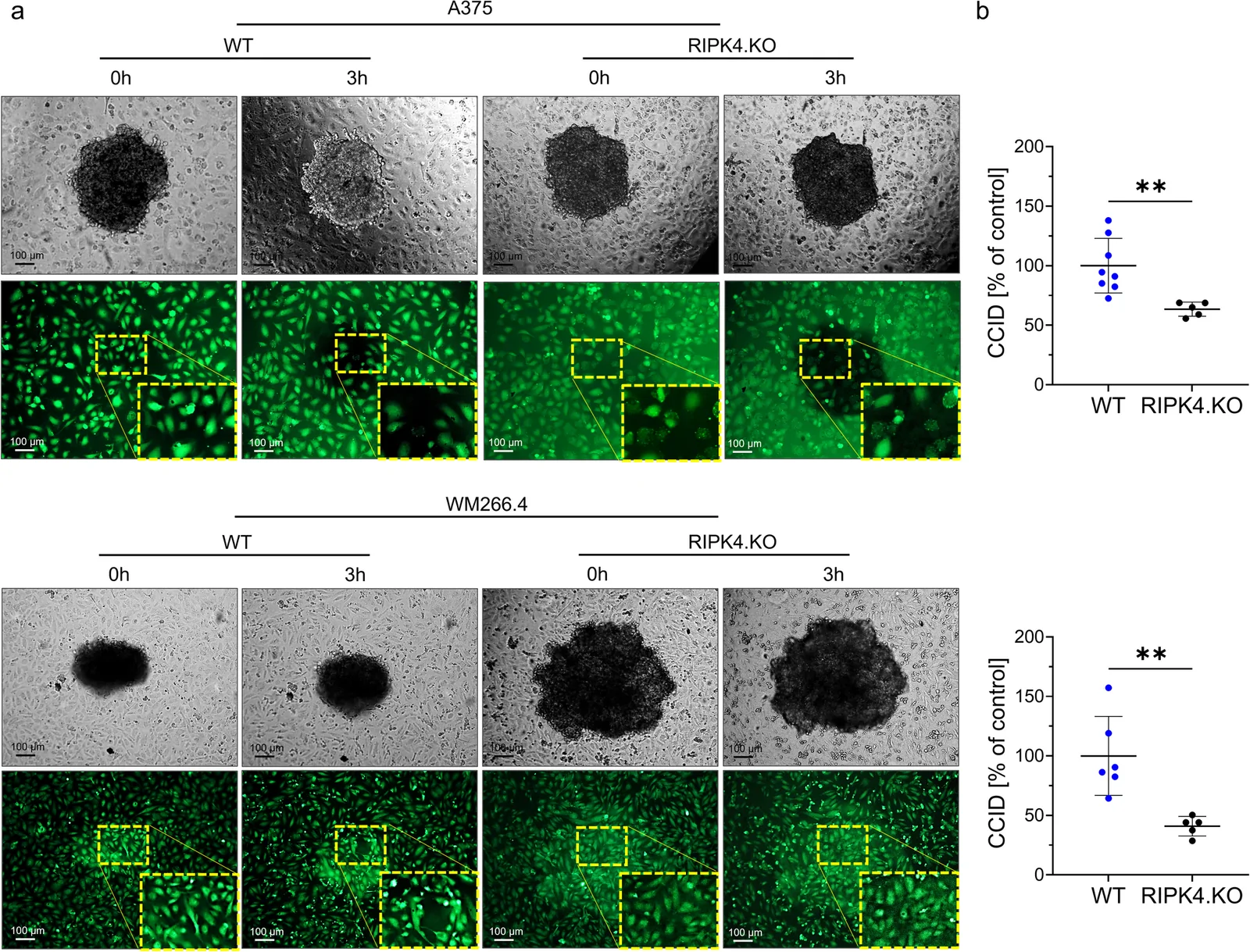
RIPK4 downregulation inhibits intravasation of A375 and WM266.4 cells. Spheroids formed from A375^RIPK4.KO^ and WM266.4^RIPK4.KO^ cells along with their respective parental wild-type (WT) lines, were placed onto a monolayer of CellTracker Green-stained HAEC cells. **(a)** Representative images of melanoma spheroids and HAEC monolayers captured in brightfield (upper panel) and HAEC cells (green, lower panel) at the indicated time points. (**b**) Quantification of circular chemorepellent-induced defects (CCIDs) in HAEC monolayers induced by A375^RIPK4.KO^, WM266.4^RIPK4.KO^ normalized to control spheroids. Data represent the mean ± SD from three independent experiments (*n* = 5–8). Statistical analysis was performed using a two-tailed unpaired Student’s *t*-test. Statistically significant differences are denoted as ** *p* < 0.01.

**Fig. 3:**
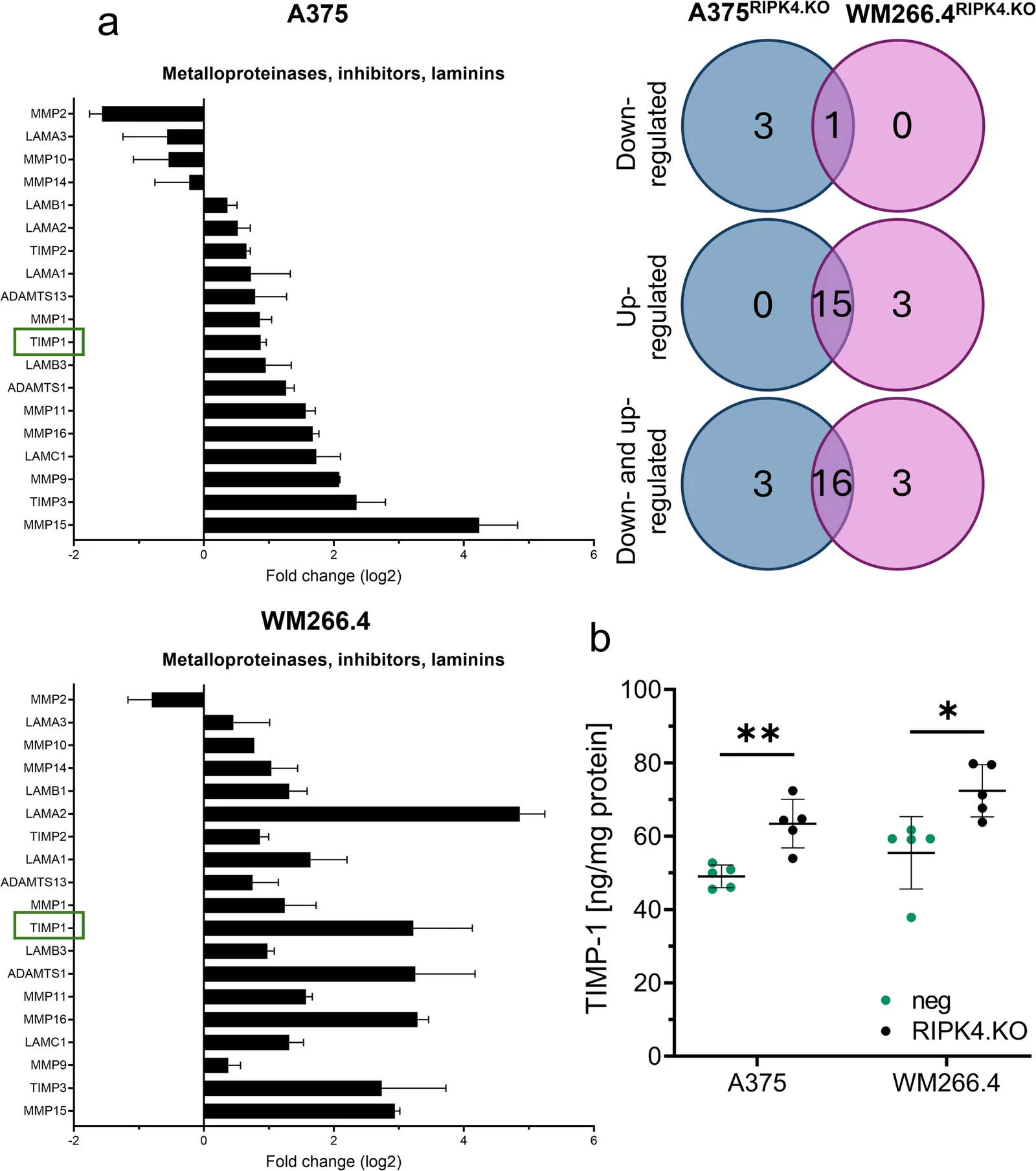
RIPK4 downregulation impairs the invasive phenotype of melanoma cells in a 3D spheroid model. (**a**) Relative mRNA expression levels of genes related to metalloproteinases, their inhibitors, and laminins were assessed in spheroids using the TaqMan™ Array Human Extracellular Matrix & Adhesion Molecules panel. Data are presented as fold change relative to the negative control and represent the mean of two independent experiments (6 spheroids pooled per sample). Results are also visualized as Venn diagrams showing the number of genes that were exclusively upregulated, downregulated, or altered in both A375^RIPK4.KO^ (blue) and WM266.4^RIPK4.KO^ (pink) cells. (**b**) TIMP-1 secretion levels in culture media of A375^RIPK4.KO^ and WM266.4^RIPK4.KO^ spheroids and their controls measured by ELISA. Data represent the mean ± SD of five independent experiments (medium from 15 pooled spheroids per sample). Statistical analysis was performed using a two-tailed unpaired Student’s *t*-test. Statistically significant differences are denoted as * *p* < 0.05, ** *p* < 0.01.

In our previously published work, RIPK4 knockout cells were found to display impaired motility and invasion, both in vitro and in vivo, as well as inhibited xenograft growth^7–9^, through the deregulation of their adhesion status^8^. The process of metastasis requires the migration and invasion of tumor cells driven by the regulated formation of adhesion structures such as focal adhesions and invasive structures such as invasive protrusions. The effect of RIPK4 on this cellular mechanism has not been fully elucidated. We therefore tested how the absence of RIPK4 affects the ability to form invasive protrusions by human melanoma cell lines (A375 and WM266.4) with distinct characteristics. A375 cells are generally considered less metastatic and are often used in studies focused on primary melanoma, while WM266.4 cells are known for their higher metastatic potential with a mesenchymal morphology and are used in research exploring the later stages of melanoma progression. We analyzed formation of invasive protrusionsafter twenty-four hours and found that, in contrast to wild-type melanoma cells (WT) and their negative control, spheroids formed from RIPK4 knockout cells failed to form invasive protrusions in both cells model (Fig. 1a-b). We next performed a circular chemorepellent-induced defects (CCID) assay using primary human aortic endothelial cells (HAECs) and melanoma spheroids. In this assay, WT melanoma cells were used instead of GFP-expressing negative control cells to allow visualization, as WT cells did not differ from the negative control in formation of invasive protrusions. Since melanoma cells disseminate from the primary tumor via the blood and lymphatic systems, this assay mimics the “entry gate” through which tumor cells intravasate into the circulation. We observed that CCID formation in HAEC monolayers was significantly reduced in RIPK4^KO^ spheroids by approximately 37% for A375 cells (*p* < 0.01) and 59% for WM266.4 cells (*p* < 0.01) (Fig. 2a-b). We next examined the expression of invasion-related genes using the TaqMan™ Human Extracellular Matrix & Adhesion Molecules Array. Among the analyzed genes, MMP-2 was the only one significantly downregulated by 66% in A375^RIPK4.KO^ and by 41% in WM266.4^RIPK4.KO^ cells (Fig. 3a). In contrast, RIPK4 depletion led to a marked upregulation of MMP inhibitors, including TIMP-1, TIMP-2, and TIMP-3. To validate these findings at the protein level, TIMP-1 secretion was assessed by ELISA (Fig. 3b), confirming significantly increased levels in the culture media of RIPK4^KO^ spheroids: by 29% in A375^RIPK4.KO^ (*p* < 0.01) and by 31% in WM266.4^RIPK4.KO^ (*p* < 0.05).

### RIPK4 deficiency impairs the bioenergetic profile of melanoma cells

**Fig. 4:**
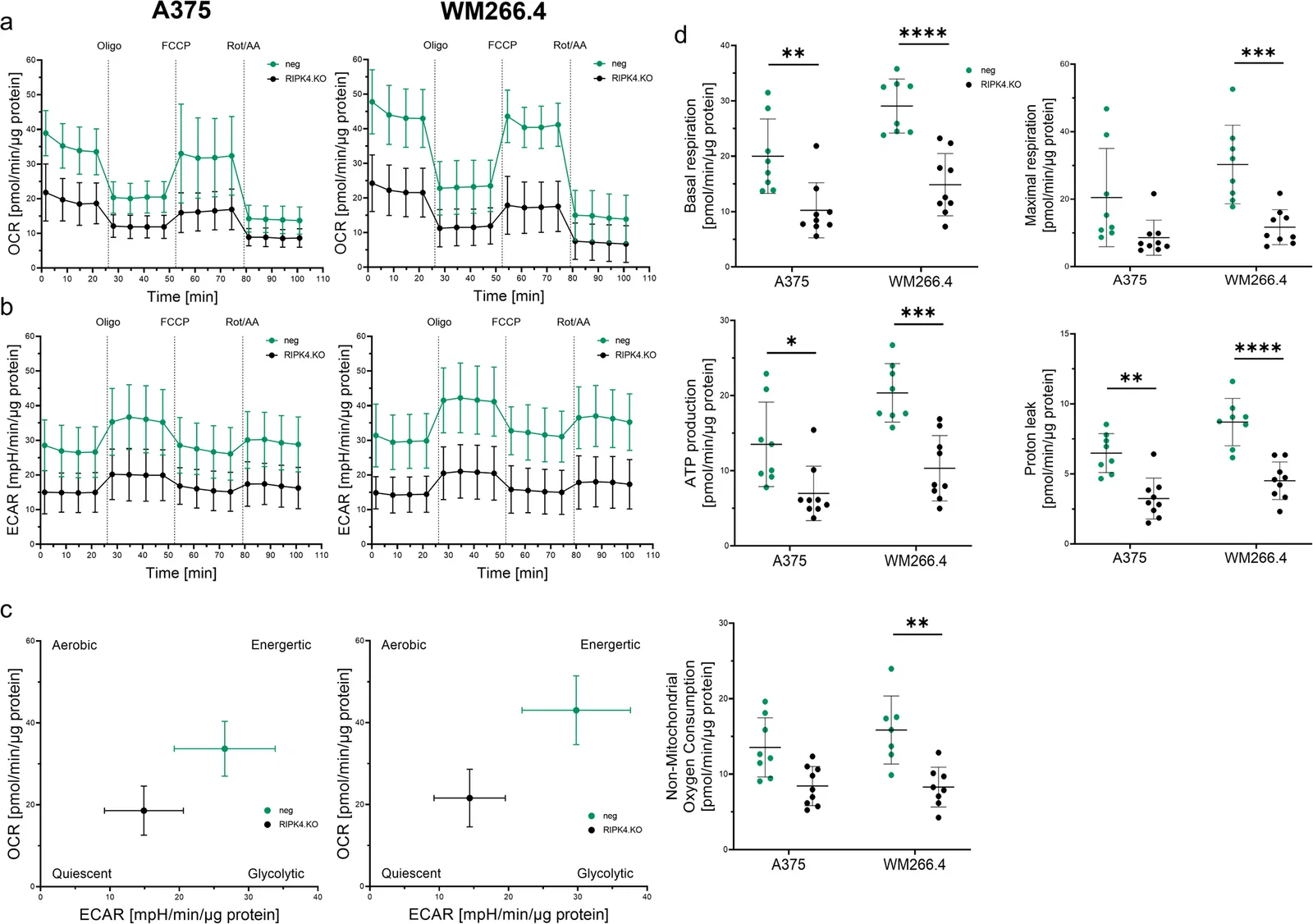
RIPK4 knockout impairs mitochondrial respiration and alters glycolytic activity in melanoma cells. Mitochondrial and glycolytic functions were assessed in WM266.4^RIPK4.KO^, and A375^RIPK4.KO^ cells and their negative controls using the Seahorse XFe96 Bioanalyzer. **(a)** Oxygen consumption rate (OCR) and **(b)** extracellular acidification rate (ECAR) were monitored in real time and normalized to baseline (pre-injection = 100%). Sequential injections of oligomycin (a complex V inhibitor, 1 µM), FCCP (a protonophore, 1 µM), and rotenone/antimycin A (complex I/III inhibitors, 1 µM each) were used to interrogate mitochondrial function. **(c)** Energy phenotype maps show the metabolic shift in RIPK4-deficient cells. **(d)** Quantitative analysis of basal respiration, maximal respiration, ATP production, proton leak, and non-mitochondrial oxygen respiration. Data represent mean ± SD from three independent experiments (*n* = 8–9). Statistical analysis was performed using a two-tailed unpaired Student’s *t*-test (* *p* < 0.05, ** *p* < 0.01, *** *p* < 0.001, **** *p* < 0.0001). OCR and ECAR profiles are displayed as relative values normalized to baseline (pre-injection = 100%) to enable comparison of dynamic metabolic responses. All bioenergetic parameters were calculated from raw OCR values and normalized to total protein content prior to statistical analysis.

To assess whether metabolic alterations contribute to the reduced invasiveness of A375^RIPK4.KO^ and WM266.4^RIPK4.KO^ cells, we simultaneously monitored their mitochondrial and glycolytic activity. Oxygen consumption rate (OCR) and extracellular acidification rate (ECAR) were measured in real time using the Seahorse XFe96 bioanalyzer. To determine functional metabolic changes, we evaluated OCR using the Seahorse XF Mito Stress Test The sequential addition of oligomycin, FCCP, and rotenone/antimycin A allowed discrimination between mitochondrial and non-mitochondrial respiration. Basal respiration rates of A375^RIPK4.KO^ and WM266.4^RIPK4.KO^ cells were lower than controls, with significant reduction in A375^RIPK4.KO^ cells (*p* < 0.01) and WM266.4^RIPK4.KO^ (*p* < 0.0001) (Fig. 4a-d). RIPK4 knockout lowered both OCR and ECAR together with ATP levels and glucose uptake, and energy mapping indicated a shift to a low-energy/quiescent phenotype (Fig. 4c). Maximal respiration was significantly reduced in WM266.4^RIPK4.KO^ cells (*p* < 0.001). Proton leak was decreased in both cell lines (A375 *p* < 0.01; WM266.4 *p* < 0.0001).

**Fig. 5:**
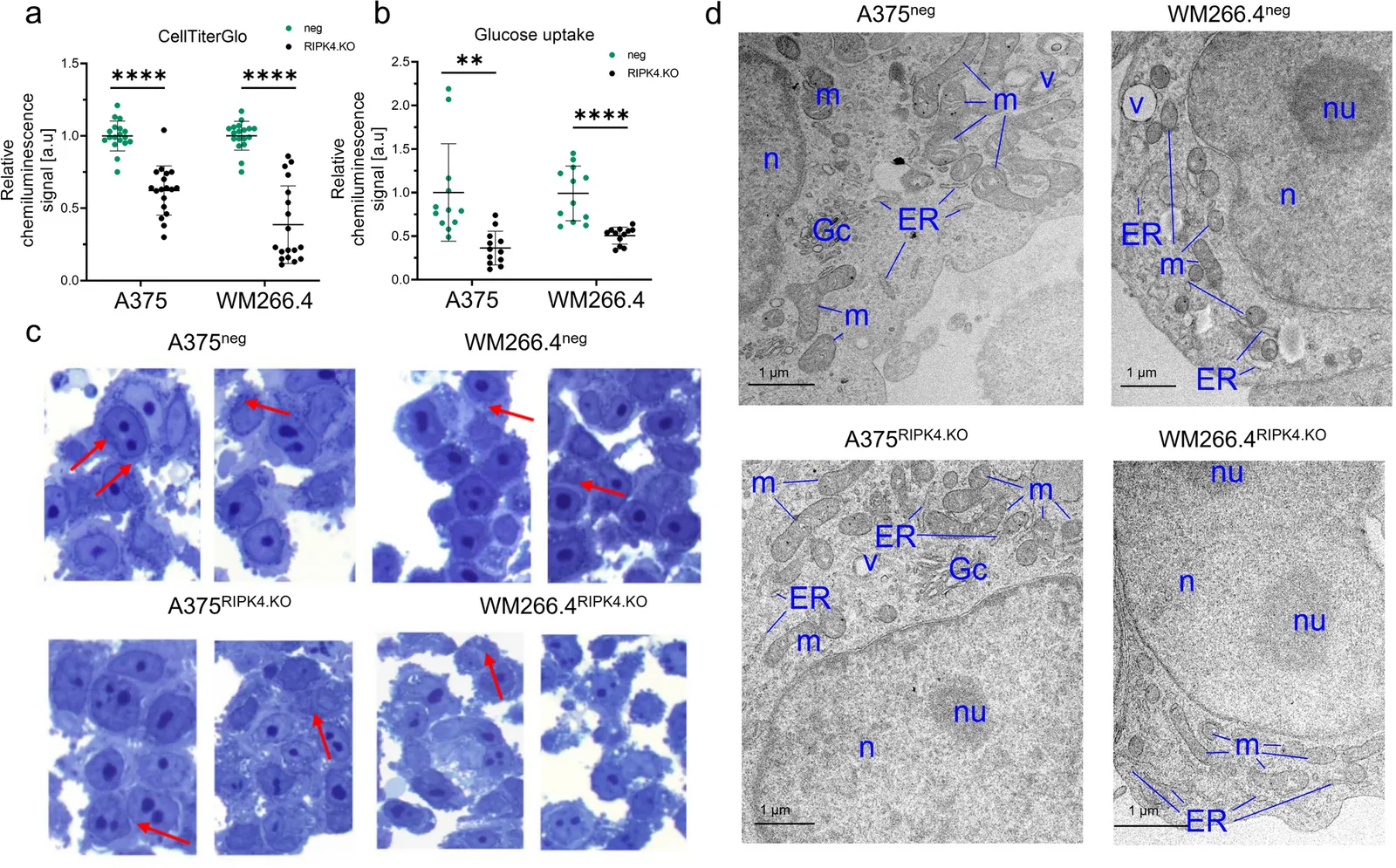
RIPK4 knockout alters metabolic activity but does not affect mitochondrial number or ultrastructure in melanoma spheroids. (**a**) ATP production assessed using the CellTiter-Glo^®^ 3D Cell Viability Assay. Data represent mean ± SD from three independent experiments, *n* = 18. (**b**) Glucose uptake measured using the Glucose Uptake-Glo™ Assay Kit. Data represent mean ± SD from three independent experiments, *n* = 12. Statistical analysis was performed using a two-tailed unpaired Student’s t-test. Statistically significant differences are denoted as ** *p* < 0.01, **** *p* < 0.0001. (**c**) Morphology of individual spheroid-forming cells showing large nuclei with well-developed nucleoli. Dense clusters are visible on one side of the nuclei (red arrows). (**d**) TEM images depicting ultrastructure of individual spheroid-forming cells. Labels: (n) nucleus, (nu) nucleoli, (m) mitochondria, (RER) rough endoplasmic reticulum, (Gc) Golgi complexes, (v) vesicles of varying size and shape.

The metabolic phenotype map revealed that A375^RIPK4.KO^ and WM266.4^RIPK4.KO^ cells adopted a more quiescent phenotype and showed a shift towards aerobic state, compared to control cells (see Fig.S2). These changes were accompanied by reduced indicators of mitochondrial oxidative phosphorylation. To complement these observations, total ATP production was assessed using an independent Seahorse-based assay. ATP production was significantly reduced in both RIPK4^KO^ cell lines (Fig. 5a), with a stronger effect observed in WM266.4 cells. Glucose uptake was decreased by approximately 64% in A375^RIPK4.KO^ (*p* < 0.01) and 50% in WM266.4^RIPK4.KO^ (*p* < 0.0001) (Fig. 5b).

### RIPK4 deletion does not affect mitochondrial architecture or number in melanoma spheroid cells

Mitochondria play a central role in cellular energy metabolism, and their abundance is often correlated with metabolic activity. Therefore, we used TEM to study mitochondrial number and ultrastructure in spheroid-forming cells. No significant differences in mitochondrial structure or number were observed between RIPK4^KO^ and control cells (Fig. 5c-d). Cells in both conditions displayed preserved nuclear morphology and similar cytoplasmic organization, with mitochondria present in comparable abundance and organization. These findings indicate that metabolic changes are not due to structural mitochondrial damage.

### Genes and proteins involved in metabolic changes under RIPK4.KO

**Fig. 6:**
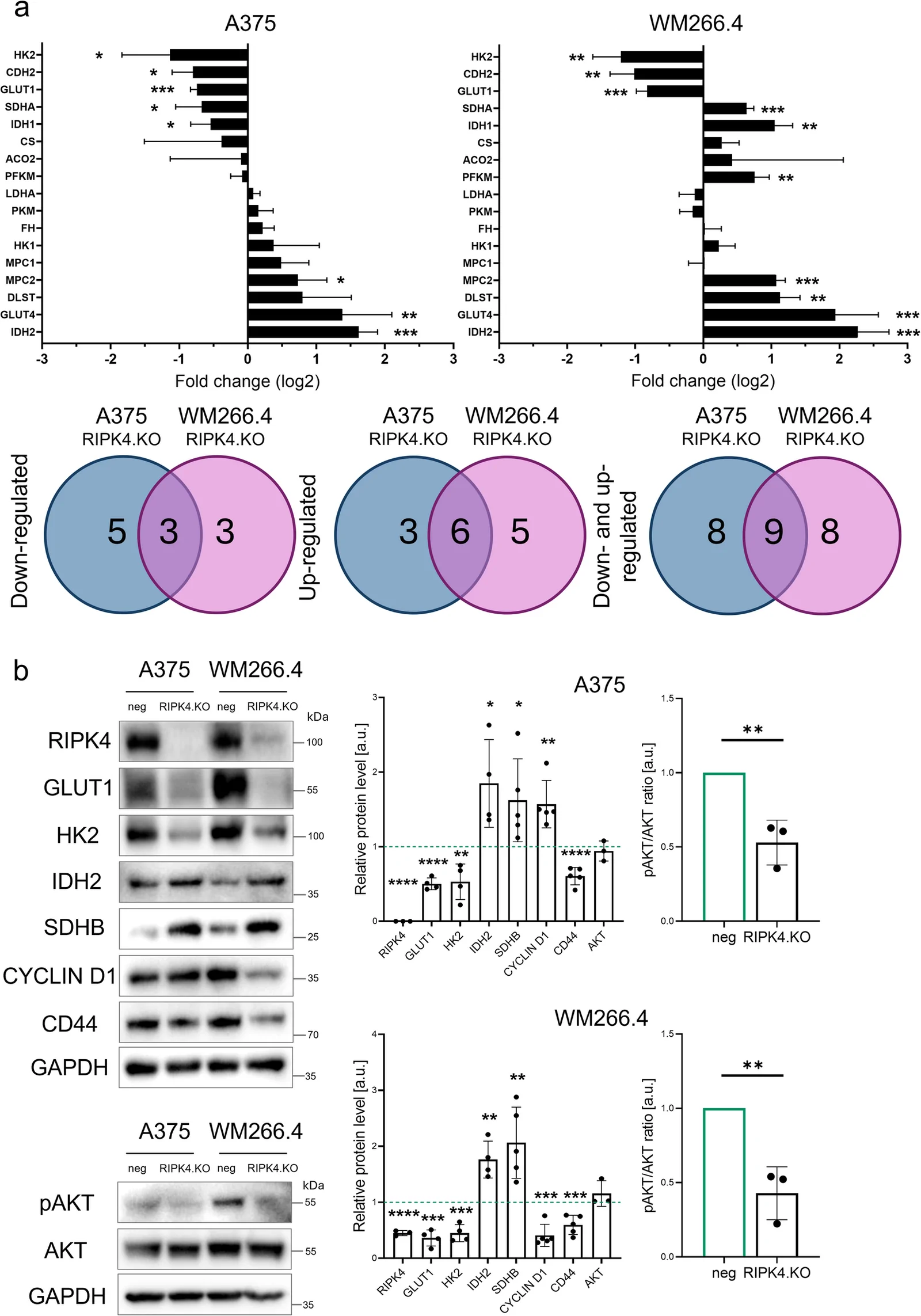
RIPK4 knockout alters expression of metabolism-related genes and proteins in 3D melanoma spheroids. Transcript (**a**) and protein (**b**) levels of metabolism-associated markers were assessed in A375 ^RIPK4.KO^ and WM266.4^RIPK4.KO^ spheroids relative to negative controls. mRNA was normalized to GAPDH (6 pooled spheroids per sample; 3–5 independent experiments). Protein levels were quantified by Western blot and densitometry, using GAPDH as a loading control (10 pooled spheroids; 3–5 independent experiments).Venn diagrams illustrate the number of genes exclusively upregulated, downregulated, or both. Circles represent A375^RIPK4.KO^ (blue) and WM266.4^RIPK4.KO^ (pink) cells. Data are mean ± SD. Statistical significance: * *p* < 0.05, ** *p* < 0.01, *** *p* < 0.001, **** *p* < 0.0001.

To further investigate the metabolic changes in RIPK4^KO^ cells, we performed a quantitative analysis of genes associated with cellular metabolism. CD44 was included as a marker of invasive potential to complement the metabolic analysis, given its established role in melanoma cell adhesion and migration. The analysis revealed differential expression of multiple metabolic genes between RIPK4^KO^ and negative control cells in both cell lines (Fig. 6a). Isocitrate dehydrogenase 2 (IDH2) expression was increased by 3-fold in A375^RIPK4.KO^ and 5-fold in WM266.4^RIPK4.KO^ cells. Similarly, GLUT4 expression was elevated by 3-fold in A375^RIPK4.KO^ cells and by 4-fold in WM266.4^RIPK4.KO^ cells compared to controls. In contrast, hexokinase 2 (HK2) expression was significantly downregulated by 51% in A375 ^RIPK4.KO^ and 56% in WM266.4 ^RIPK4.KO^, while GLUT1 expression was reduced by 40% in A375^RIPK4.KO^ and by 43% in WM266.4^RIPK4.KO^ cells. Protein analysis confirmed these transcriptomic changes (Fig. 6b). IDH2 protein levels were increased by 84% in A375^RIPK4.KO^ (*p* < 0.05) and by 90% in WM266.4^RIPK4.KO^ cells (*p* < 0.01). Conversely, GLUT1 levels were reduced by 56% in A375^RIPK4.KO^ (*p* < 0.0001) and by 64% in WM266.4^RIPK4.KO^ cells (*p* < 0.001), while HK2 protein levels decreased by 47% in A375^RIPK4.KO^ (*p* < 0.01) and by 55% in WM266.4^RIPK4.KO^ cells (*p* < 0.001). Additionally, SDHB protein levels (a subunit of complex II) increased by 62% in A375^RIPK4.KO^ (*p* < 0.05) and by twofold in WM266.4^RIPK4.KO^ cells (*p* < 0.01) alongside other metabolic alterations observed in RIPK4^KO^ cells. This metabolic shift was accompanied by a 59% reduction in cyclin D1 in WM266.4^RIPK4.KO^ cells (*p* < 0.001).

### RIPK4 regulates AKT signaling in 3D melanoma models

The PI3K/AKT signaling pathway is a central regulator of glucose metabolism, controlling glucose uptake and glycolytic enzyme activity. Activated AKT promotes GLUT1 expression and supports glycolytic flux through downstream metabolic regulators, including key glycolytic enzymes such as HK2 and mTOR-dependent anabolic signaling. Elevated levels of phosphorylated AKT (p-AKT) have been associated with increased GLUT1 expression and enhanced glucose uptake in multiple malignancies^18,19^. Importantly, previous work by Madej et al.^10^ demonstrated that RIPK4 downregulation suppresses FAK/AKT signaling in melanoma cells, indicating a functional link between RIPK4 and this pathway. In line with these findings, we observed a substantial reduction of p-AKT levels under 3D culture conditions in RIPK4^KO^ cells approximately 47% in A375 (*p* < 0.01) and 57% in WM266.4 (*p* < 0.01) compared with control spheroids (Fig. 6b). These results indicate that RIPK4 is associated with regulation of AKT signaling in 3D melanoma models.

### RIPK4 rescue restores AKT signaling and glycolytic markers but not metabolic flux in A375 cells

**Fig. 7:**
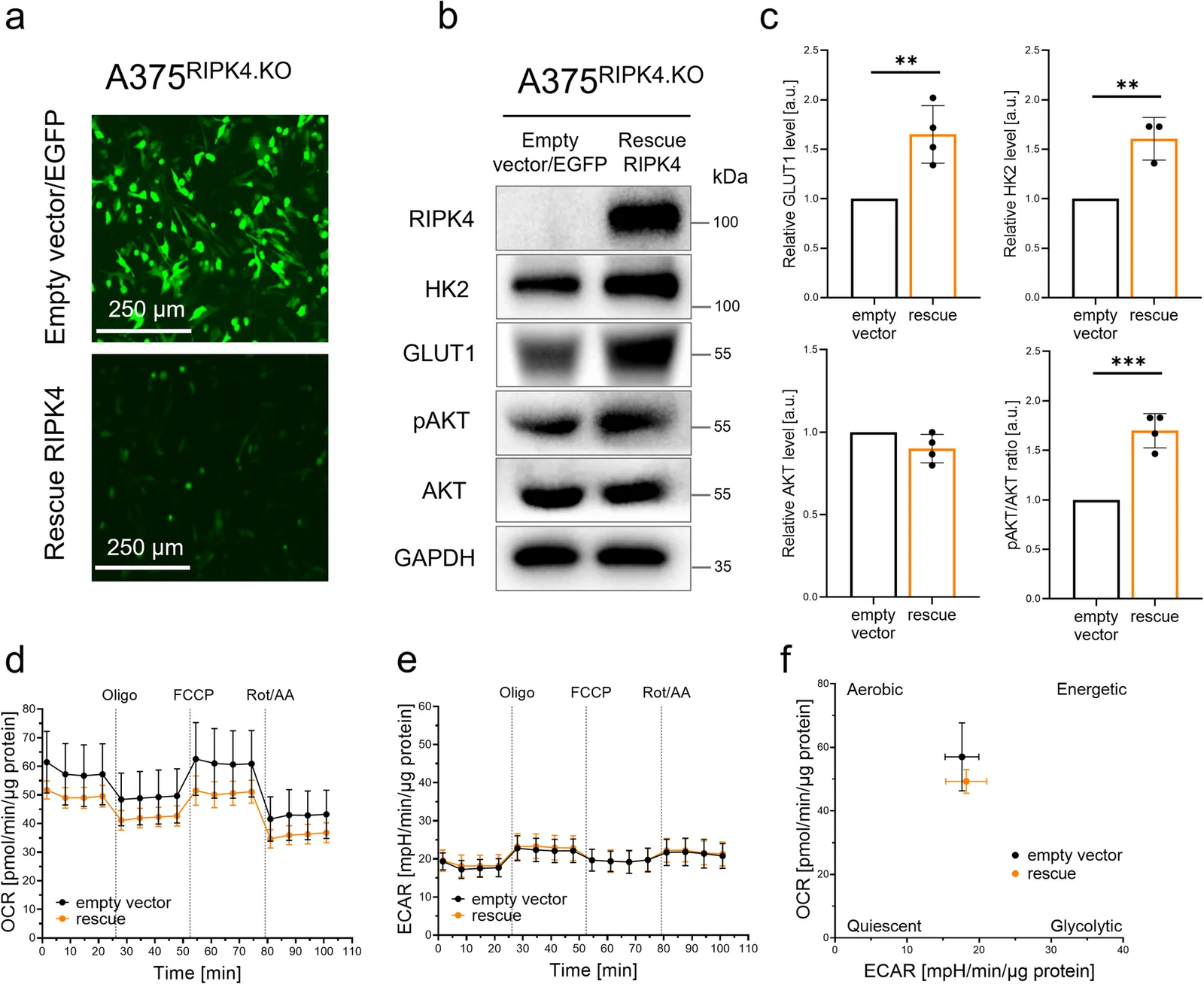
RIPK4 rescue restores protein levels without fully recovering metabolic activity. (**a**) Representative GFP fluorescence images showing successful transfection of RIPK4 in A375^RIPK4.KO^ cells. (**b**) Western blot analysis of p-AKT, AKT, GLUT1, and HK2 levels in RIPK4^KO^ cells following RIPK4 re-expression (rescue) compared with negative control (empty vector) in 2D, GAPDH was used as a loading control. (**c**) Densitometric quantification is presented as mean ± SD from three independent experiments, *n* = 3–4. Statistical analysis was performed using a two-tailed unpaired Student’s t-test. Significant differences are indicated as ** *p* < 0.01, and *** *p* < 0.001. (**d**) Oxygen consumption rate (OCR) and **(e)** extracellular acidification rate (ECAR) were monitored in real time. Measurements were normalized to baseline (pre-injection) values set at 100%, followed by sequential injections of oligomycin (a complex V inhibitor, 1 µM), FCCP (a protonophore, 1 µM), and rotenone/antimycin A (complex I/III inhibitors, 1 µM each). **(f)** Energy phenotype maps show no significant difference in RIPK4^rescue^ cells. *n* = 3.

To assess whether the observed metabolic and signaling changes were directly due to RIPK4 loss, rescue experiments were performed in A375^RIPK4.KO^ cells (which carry a complete RIPK4 deletion) by re-expressing RIPK4. Restoration of RIPK4 led to a marked increase in AKT phosphorylation, approximately twofold higher than in RIPK4^KO^ cells (Fig. 7a-c). Consistent with AKT reactivation, expression of glycolytic markers was significantly elevated with both GLUT1 and HK2 protein levels increasing nearly twofold following RIPK4 re-expression. However, despite the restoration of p-AKT and glycolytic protein expression, functional metabolic analysis revealed no recovery of glycolytic flux. Seahorse measurements showed that extracellular acidification rates (ECAR) and oxygen consumption rates (OCR) remained comparable to RIPK4^KO^ cells (Fig. 7d-f).

Thus, reactivation of AKT signaling and increased expression of GLUT1 and HK2 were insufficient to restore metabolic function. These findings indicate that RIPK4-dependent metabolic regulation cannot be fully explained by AKT-GLUT1 signaling alone and may involve longer-term metabolic adaptations that are not rapidly reversible upon short-term re-expression.

### GLUT1 Expression in Lung Metastases

**Fig. 8:**
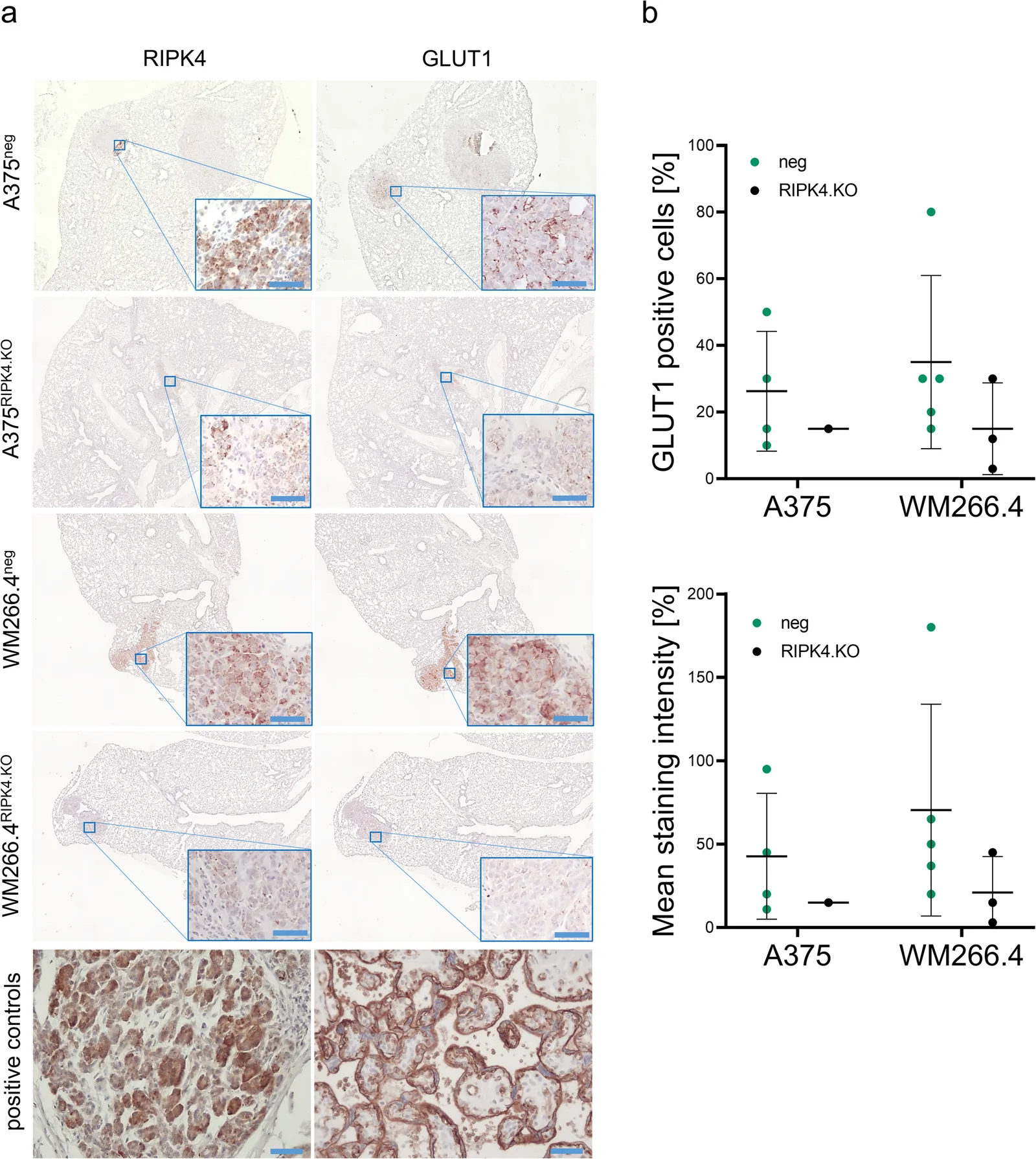
Immunohistochemical analysis of GLUT1 and RIPK4 in murine lung metastases. (**a**) Representative immunohistochemical staining of GLUT1 and RIPK4 in lung sections from NOD/SCID mice intravenously injected with A375 and WM266.4 cells (negative control and RIPK4.KO), along with positive controls (placenta for GLUT1 and pancreas for RIPK4). Scale bar = 50 μm. (**b**) Quantification of GLUT1 expression in A375^neg^ (*n* = 4), A375^RIPK4.KO^ (*n* = 1), WM266.4^neg^ (*n* = 5), and WM266.4^RIPK4.KO^ (*n* = 3). Percentage of GLUT1-positive cells (upper panel). Mean staining intensity (score 0–3) relative to the respective positive controls (lower panel).

To extend our in vitro observations, we evaluated GLUT1 and RIPK4 expression in metastatic lesions using formalin-fixed, paraffin-embedded murine lung tissues derived from a previously published the study Immunohistochemical analysis (Fig. 8a-b) was performed to quantify the proportion of GLUT1-positive cells and mean staining intensity. Placenta (GLUT1) and pancreas (RIPK4) served as positive controls, confirming antibody specificity and staining reliability. GLUT1 expression was detectable in metastatic foci in both models. The predominant localization pattern was discontinuous membranous staining, observed in 83% of WM266.4-derived lung lesions and 75% of A375-derived lesions. RIPK4 knockout was associated with reduced GLUT1 immunoreactivity. In WM266.4-derived metastases, RIPK4 loss resulted in a decreased proportion of GLUT1-positive cell and lower staining intensity compared with control lesions. In the A375 model, only a single RIPK4.KO specimen was available; however, a similar trend toward reduced GLUT1 staining was observed. Given the limited sample size in the A375^RIPK4.KO^ group, these findings should be interpreted as descriptive rather than quantitative.

Collectively, these in vivo data are consistent with our in vitro protein analyses demonstrating reduced GLUT1 expression upon RIPK4 depletion and support an association between RIPK4 and GLUT1 expression in melanoma metastasis.

## Discussion

This study indicates that RIPK4 is associated with both metabolic regulation and invasive capacity in melanoma cells. Our findings provide evidence that RIPK4 depletion affects melanoma metabolism, suggesting a potential link between metabolic state and invasion. This was demonstrated by: (i) impaired bioenergetics in RIPK4^KO^ melanoma cells (as measured by Seahorse and ATP analysis); (ii) differences in transcript and protein levels associated with metabolism, such as increased IDH2 and GLUT4 levels and decreased HK2 and GLUT1 levels compared to control cells of both lines; and (iii) inhibited invasion and intravascular invasion of RIPK4^KO^ cells. In addition, RIPK4^KO^ cells exhibited increased expression of SDHB in both melanoma cell lines, consistent with a shared metabolic adaptation. In contrast, cyclin D1 levels were reduced only in WM266.4^RIPK4.KO^ cells, while remaining unchanged in A375^RIPK4.KO^ cells, indicating cell line-specific differences in cell cycle regulation. Although reduced cyclin D1 together with impaired glycolytic and oxidative phosphorylation activity may suggest reduced proliferative activity, the absence of a consistent effect across both models argues against a uniform quiescent-like state. Instead, these findings support a model in which RIPK4 loss induces a globally metabolically compromised phenotype, while effects on cell cycle regulation are context-dependent. Given that cyclin D1 is regulated by both Wnt/β-catenin and AKT/mTOR signaling pathways, the observed heterogeneity may reflect intrinsic differences in baseline pathway activity between WM266.4 and A375 cells, or differential coupling between metabolic and proliferative signaling downstream of RIPK4. Functionally, these changes were accompanied by markedly inhibited formation of invasive protrusions and impaired invasion potential.

RIPK4 has emerged as a context-dependent regulator of tumor progression, playing distinct roles in different cancer types. In melanoma and epithelial-derived solid tumors such as pancreatic, bladder, and ovarian cancers, RIPK4 has been shown to enhance epithelial -mesenchymal transition (EMT), promote cell motility, and correlate with poor prognosis^6,20^.

In contrast, in squamous cell carcinomas of the skin and tongue, RIPK4 appears to act as a tumor suppressor^21^. Despite these advances, the metabolic role of RIPK4 has remained unexplored until now. Given that RIPK4 has been implicated in multiple signaling pathways, including NF-κB, Wnt/β-catenin, and FAK signaling, the observed phenotype likely reflects integrated effects across several pathways rather than a single linear AKT-GLUT1 mechanism.

Invasive protrusions are exclusive to tumour cells and play a key role in cancer progression. The primary function of invasive protrusionsis to facilitate tumour cell invasion by degrading the extracellular matrix (ECM), enabling entry into blood vessels and promoting metastasis. These structures usually form on the side of the cell that is next to the basement membrane. There, they localise and secrete MMPs (including MT1-MMP, MMP2 and MMP9) at the tips of the protrusions, which promotes ECM degradation^22^. We found that RIPK4^KO^ cells failed to form invasive protrusions (A375 and WM266.4), which coincided with reduced invasion, downregulation of MMP‑2 and increased TIMP‑1 expression/secretion. Consistently, RIPK4 silencing via siRNA reduced invasive potential and downregulated pro-invasive proteins including MMP2, MMP9, and N-cadherin^9^. Similar observations were reported in cervical cancer cells, where RIPK4 knockdown decreased migration and invasion by downregulating vimentin, fibronectin, and MMP2^21^.

It is well known that cell movement is powered by ATP energy, and that migration is bioenergetically demanding^23,24^. Consistent with this, we demonstrated that RIPK4 deletion reduces ATP production, glucose uptake, and basal oxygen consumption in melanoma cells. Seahorse energy mapping revealed that RIPK4^KO^ cells shift toward a metabolically less active state. Interestingly, we found that RIPK4^KO^ melanoma cells shift to a low-energetic and aerobic bioenergetic profile, indicating that the cell utilizes predominantly mitochondrial respiration. In contrast, RIPK4-positive melanoma cells display a glycolytic bioenergetic profile, which is consistent with existing literature. Thus, melanoma cells rely heavily on glycolysis, the breakdown of glucose for energy, even when oxygen is abundant.

Melanoma is characterized by profound metabolic reprogramming, which includes increased glucose uptake and a marked dependence on glycolysis, even under normoxic conditions. This metabolic phenotype supports tumor growth, proliferation, and survival by ensuring a constant supply of ATP and biosynthetic intermediates necessary for rapid cell division^25^. Glycolysis and oxidative phosphorylation (OXPHOS) are the two principal energy-producing pathways in cancer cells. Notably, most tumors, including melanoma, retain the metabolic plasticity to switch between these two pathways depending on environmental cues such as nutrient availability or oxygen levels^26^.

In RIPK4^KO^ melanoma cells, we observed a global reduction in bioenergetic activity affecting both glycolysis and mitochondrial respiration, rather than a shift toward oxidative metabolism. This shift is associated with specific molecular alterations, including downregulation of hexokinase 2 (HK2) - a key glycolytic enzyme and upregulation of isocitrate dehydrogenase 2 (IDH2), a mitochondrial enzyme essential for the tricarboxylic acid (TCA) cycle and oxidative energy production.

HK2 has been previously implicated in promoting the metastatic potential of melanoma cells^27^, and its downregulation in RIPK4^KO^ cells may contribute to reduced tumor aggressiveness and energy production. Consistent with this, we also observed decreased expression of GLUT1 at both transcript and protein levels. GLUT1, along with GLUT3, is frequently upregulated in malignant melanoma compared to benign nevi, and its high expression correlates with tumor aggressiveness and poor clinical^28,29^. GLUT1 belongs to the glucose transporter (GLUT) family and facilitates the uptake of glucose, a key substrate for both glycolysis and OXPHOS^30^. Thus, downregulation of both GLUT1 and HK2 in RIPK4^KO^ melanoma cells leads to diminished glucose uptake and reduced glycolytic flux. To complement the experimental findings, correlation analysis using the GEPIA platform demonstrated a significant positive association between RIPK4 and HK2 expression in melanoma datasets, whereas correlations with IDH2 and SLC2A1 were weak or not statistically significant (Fig.S3). Our results further indicate that oxidative phosphorylation is also compromised in RIPK4-deficient cells, as evidenced by reduced basal oxygen consumption. Since glucose uptake and OXPHOS are interdependent processes essential for efficient ATP production, the concurrent impairment of both pathways in RIPK4^KO^ cells suggests a global decline in bioenergetic capacity. Together, these metabolic measurements motivated a mechanistic analysis of AKT‑linked glycolytic control and invasion.

Consistent with reduced mitochondrial function, RIPK4^KO^ melanoma cells exhibited decreased proton leak in both A375 and WM266.4 cell lines. Proton leak refers to the movement of protons across the inner mitochondrial membrane independent of ATP synthase activity. Although often considered a form of energy dissipation, regulated proton leak serves important roles in minimizing oxidative stress and fine-tuning ATP production. A decrease in proton leak, as observed in RIPK4-deficient cells, may suggest enhanced coupling efficiency of the electron transport chain, allowing more efficient ATP generation with reduced energy waste. This phenomenon could represent a compensatory mechanism to sustain cellular energy levels despite impaired glycolytic input. Increased SDHB and IDH2 may reflect an adaptive adjustment at the level of respiratory‑chain/TCA components without a net gain in flux. On this background, our data suggest that RIPK4 influences both adhesion‑associated processes and metabolic regulation, linking these phenotypes at a functional level via FAK/AKT signaling, RIPK4 maintains a glycolytic program (GLUT1, HK2) that supports formation of invasive protrusions and intravasation.

Transmission electron microscopy (TEM) analysis supported this interpretation by revealing that mitochondrial number and ultrastructural architecture remained intact in RIPK4^KO^ cells, suggesting that changes in mitochondrial function are not due to gross mitochondrial damage or loss, but rather due to altered metabolic regulation.

Interestingly, although A375 and WM266.4 melanoma cells differ in their metastatic potential and baseline phenotype, they share similar metabolic profiles both are energetically active and exhibit glycolytic dominance under normal conditions. The consistent metabolic reprogramming observed upon RIPK4 knockout across both cell lines suggests that RIPK4 may serve a central role in regulating melanoma bioenergetics, irrespective of intrinsic differences. These data suggest that RIPK4 may represent a potential therapeutic target that warrants further validation in more comprehensive in vivo models. Our mechanistic analyses indicate that RIPK4-dependent metabolic regulation is at least partially mediated via AKT signaling. In 3D spheroids, RIPK4 deficiency resulted in reduction of p-AKT levels. Given the role of the PI3K/AKT pathway in regulating GLUT1 expression and glucose metabolism, these findings suggest a potential link between RIPK4 and glycolytic regulation. The observed reductions in GLUT1 and HK2 expression in RIPK4^KO^ cells are therefore consistent with attenuation of AKT-driven metabolic programming. Immunohistochemical analysis of lung metastases provided supportive evidence of reduced GLUT1 expression in RIPK4-deficient tumors in vivo. However, these findings are based on immunohistochemical analysis and should be interpreted with caution, as they do not provide direct functional insight into metabolic activity in vivo. Although AKT activation could not be assessed due to limited tissue availability, both the percentage of GLUT1-positive cells and mean staining intensity were lower in RIPK4^KO^-derived lesions compared to controls. The predominant discontinuous membranous pattern of GLUT1 localization suggests dynamic regulation of glucose transporter trafficking, a process often associated with aggressive tumor phenotypes. These data support the notion that RIPK4 contributes to the maintenance of a glycolytic phenotype not only in vitro but also within the metastatic microenvironment. Rescue experiments refined this mechanistic model. Re-expression of RIPK4 in A375^RIPK4.KO^ cells restored AKT phosphorylation approximately twofold compared with knockout cells and nearly doubled GLUT1 and HK2 protein expression. However, metabolic flux measured by Seahorse analysis remained comparable to RIPK4-deficient cells, indicating that recovery of AKT signaling and glycolytic markers is insufficient to fully restore glycolytic capacity. This uncoupling between restoration of AKT signaling and glycolytic markers and the lack of recovery of metabolic flux suggests that RIPK4-dependent metabolic regulation cannot be explained solely by AKT-GLUT1 signaling. One possible explanation is the presence of metabolic adaptation or “metabolic memory,” where prolonged RIPK4 loss induces bioenergetic rewiring that is not readily reversible upon short-term re-expression. The lack of flux recovery after short‑term RIPK4 re‑expression, despite increased p‑AKT, GLUT1, and HK2, suggests slowly reverting adaptations rather than rapid reversibility. The observed mismatch between AKT activation, glycolytic enzyme levels, and functional metabolic output further supports the notion that RIPK4-dependent metabolic regulation cannot be attributed solely to AKT signaling. Instead RIPK4 may integrate multiple processes, including cytoskeletal dynamics, adhesion signaling, and metabolic control, thereby linking bioenergetic state with invasive behavior.

Taken together, these findings suggest that RIPK4 may function as a context-dependent regulator linking metabolic state and invasive invasive capacity in melanoma cells, rather than acting through a single linear pathway.

## Conclusion

Overall, these findings suggest that RIPK4 is associated with both metabolic regulation and invasive behavior in melanoma cells. Loss of RIPK4 induces a metabolically compromised state characterized by reduced glycolytic and mitochondrial activity, accompanied by impaired invasion. While RIPK4 re-expression restores AKT signaling and glycolytic markers, the lack of recovery of metabolic flux indicates that additional regulatory mechanisms contribute to the phenotype. These results highlight the complexity of RIPK4-dependent metabolic regulation and suggest that it may involve integrated signaling networks linking bioenergetics and invasion.

### Limitation

This study has several limitations. First, although our data demonstrate consistent associations between RIPK4 expression, AKT signaling, and metabolic alterations, we do not establish direct causality between these processes. In particular, functional experiments manipulating AKT activity or GLUT1 expression were not performed and would be required to define the mechanistic relationship.

Second, while metabolic and invasive phenotypes were observed concurrently, a direct causal link between altered bioenergetics and impaired invasion was not experimentally addressed.

Third, the in vivo analyses are based on immunohistochemical assessment of GLUT1 expression in metastatic lesions, which provides limited functional insight into metabolic activity in vivo.

Fourth, the lack of recovery of metabolic flux following RIPK4 re-expression suggests the involvement of additional regulatory mechanisms, such as metabolic adaptation or compensatory pathways, which were not explored in this study.

Finally, Seahorse metabolic flux analyses were performed in 2D monolayer cultures due to technical and cost constraints associated with 3D spheroid metabolic profiling. Since cellular metabolism is strongly influenced by microenvironmental conditions, including oxygen and nutrient gradients, this may limit direct comparison with 3D functional assays. However, the consistency of metabolic alterations observed across multiple complementary assays supports the robustness of the overall phenotype. Importantly, the relevance of metabolic plasticity in melanoma is further underscored by its established role in therapy resistance. Enhanced glycolysis and mitochondrial rewiring can fuel ATP-dependent drug efflux, maintain redox homeostasis, and support pro-survival signaling pathways^31^, highlighting the need for future studies to explore whether RIPK4-dependent metabolic regulation contributes to treatment response.

## Supplementary Information

Below is the link to the electronic supplementary material.

Supplementary Material 1

## Data Availability

The datasets generated and/or analysed during the current study are publicly available in the Jagiellonian University RODBUK repository at https://doi.org/10.57903/UJ/DBJFAB.

## Abbreviations

CCID: Circular Chemorepellent–Induced Defects
ECAR: Extracellular Acidification Rate
ECM: Extracellular Matrix
GLUT1/4: Glucose Transporter 1/4
HAEC: Human Aortic Endothelial Cells
HK2: Hexokinase 2
IDH2: Isocitrate Dehydrogenase 2
KO: Knockout
MMP: Matrix Metalloproteinases
OCR: Oxygen Consumption Rate
OXPHOS: Oxidative Phosphorylation
RIPK4: Receptor–Interacting Serine/Threonine–Protein Kinase 4
SDHB: Succinate Dehydrogenase complex iron sulfur subunit B
TCA Cycle: Tricarboxylic Acid Cycle
TEM: Transmission Electron Microscopy
TIMP: 1–Tissue Inhibitor of Metalloproteinases 1
