# Supplementary material for: RIPK4 is associated with altered bioenergetics and invasive in melanoma three-dimensional models
Source: Sci Rep. 2026 Jun 23;16:28854. doi: 10.1038/s41598-026-59563-y (PMC13578279; doi:10.1038/s41598-026-59563-y)
Supplement: Supplementary file 1 [file 41598_2026_59563_MOESM1_ESM.docx]

Scientific Reports

*Supplementary materials*

RIPK4 is associated with altered bioenergetics and invasive in melanoma three-dimensional models.

Norbert Wronski^1,2^, Jan Wolnik^2,3^, Wacław Tworzydło^4^, Aleksandra Bienia^1,2^, Justyna Gogola-Mruk^5^, Anna A Brożyna^6,7^, Anna Ptak^5^, Agnieszka Wolnicka-Glubisz^1*^

^1^	Department of Biophysics and Cancer Biology, Faculty of Biochemistry, Biophysics and Biotechnology, Jagiellonian University, Gronostajowa 7, 30-387 Krakow, Poland

^2^ Doctoral School of Exact and Natural Sciences, Jagiellonian University, Kraków, Poland

^3^	Department of Medical Biotechnology, Faculty of Biochemistry, Biophysics and Biotechnology, Jagiellonian University, Gronostajowa 7, 30-387 Krakow, Poland

^4^	Department of Developmental Biology and Invertebrate Morphology, Institute of Zoology and Biomedical Research, Jagiellonian University, Gronostajowa 9, 30-387 Krakow, Poland

^5^	Laboratory of Physiology and Toxicology of Reproduction, Institute of Zoology and Biomedical Research, Jagiellonian University, Gronostajowa 9, 30-387 Krakow, Poland

^6^ Department of Human Biology, Institute of Biology, Faculty of Biological and Veterinary Sciences, Nicolaus Copernicus University, ul. Lwowska 1, 87-100 Toruń, Poland.
^7^ Institute of Advanced Studies, Nicolaus Copernicus University in Toruń, Wileńska 4, 87-100 Toruń, Poland.

Corresponding author: Agnieszka Wolnicka-Głubisz, email: a.wolnicka-glubisz@uj.edu.pl

**Fig.S1 Original unedited blots associated with the Figure 6b (a) and Figure 7b (b) containing the Western blot results.**

**Fig.S2 Wild-type and negative control A375 and WM266.4 melanoma cells display comparable mitochondrial respiration and glycolytic activity.** Mitochondrial and glycolytic functions were assessed in wild-type (WT) and negative control A375 and WM266.4 melanoma cells using the Seahorse XFe96 Bioanalyzer. (**a**) Oxygen consumption rate (OCR) and **(b)** extracellular acidification rate (ECAR) were monitored in real time. Measurements were normalized to baseline (pre-injection) values set at 100%, followed by sequential injections of oligomycin (a complex V inhibitor, 1 µM), FCCP (a protonophore, 1 µM), and rotenone/antimycin A (complex I/III inhibitors, 1 µM each). **(c)** Energy phenotype maps show the metabolic shift in RIPK4-deficient cells.

**Fig.S3** **Correlation analysis of RIPK4 expression with selected metabolic genes in melanoma was performed using GEPIA.** Data were accessed on 23 February 2026 (<http://gepia.cancer-pku.cn>). RIPK4 expression shows a significant positive correlation with HK2, whereas correlations with IDH2 and SLC2A1 are weak or not statistically significant.
